# Exploring the Molecular Interactions of 7,8-Dihydroxyflavone and Its Derivatives with TrkB and VEGFR2 Proteins

**DOI:** 10.3390/ijms160921087

**Published:** 2015-09-03

**Authors:** Nitin Chitranshi, Vivek Gupta, Sanjay Kumar, Stuart L. Graham

**Affiliations:** 1 Faculty of Medicine and Health Sciences, Macquarie University, F10A, 2 Technology Place, North Ryde, NSW 2109, Australia; E-Mails: vivek.gupta@mq.edu.au (V.G.); stuart.graham@mq.edu.au (S.L.G.); 2 Bioinformatics Centre, Biotech Park, Lucknow, Uttar Pradesh 226021, India; E-Mail: sanjjaybharti@gmail.com; 3 Save Sight Institute, Sydney University, Sydney, NSW 2109, Australia

**Keywords:** 7,8-DHF, TrkB receptor, VEGFR2 receptor, docking, molecular dynamics, neurodegenerative disorder, retina, glaucoma, BDNF

## Abstract

7,8-Dihydroxyflavone (7,8-DHF) is a TrkB receptor agonist, and treatment with this flavonoid derivative brings about an enhanced TrkB phosphorylation and promotes downstream cellular signalling. Flavonoids are also known to exert an inhibitory effect on the vascular endothelial growth factor receptor (VEGFR) family of tyrosine kinase receptors. VEGFR2 is one of the important receptors involved in the regulation of vasculogenesis and angiogenesis and has also been implicated to exhibit various neuroprotective roles. Its upregulation and uncontrolled activity is associated with a range of pathological conditions such as age-related macular degeneration and various proliferative disorders. In this study, we investigated molecular interactions of 7,8-DHF and its derivatives with both the TrkB receptor as well as VEGFR2. Using a combination of molecular docking and computational mapping tools involving molecular dynamics approaches we have elucidated additional residues and binding energies involved in 7,8-DHF interactions with the TrkB Ig2 domain and VEGFR2. Our investigations have revealed for the first time that 7,8-DHF has dual biochemical action and its treatment may have divergent effects on the TrkB via its extracellular Ig2 domain and on the VEGFR2 receptor through the intracellular kinase domain. Contrary to its agonistic effects on the TrkB receptor, 7,8-DHF was found to downregulate VEGFR2 phosphorylation both in 661W photoreceptor cells and in retinal tissue.

## 1. Introduction

Flavonoids are a naturally occurring class of chemicals, which are abundant in fruits and vegetables and exert diverse biological effects. Recent studies have identified that a flavonoid derivative, 7,8-DHF acts as a high-affinity tropomyosin related kinase receptor B (TrkB) agonist that provokes receptor dimerization and autophosphorylation and activation of downstream signalling *in vivo* [1]. This compound has been shown to be highly neuroprotective in several disease conditions such as Alzheimer’s disease [2], Parkinson’s disease [1], Rett syndrome [3], and Huntington’s disease [4]. It can readily penetrate the blood–brain barrier and is bioavailable orally [5]. We have shown that 7,8-DHF can play a role in the protection of retinal ganglion cells from excitotoxicity and oxidative stress mediated degeneration [6]. TrkB is a receptor tyrosine kinase which is well expressed in retina and is important in the development of the inner retinal network [6,7]. 7,8-DHF can activate the TrkB receptor several fold and can induce the activation of downstream pro-survival signalling cascades such as Akt and MAPK/Erk pathways. While several studies have shown that neuroprotective actions of 7,8-DHF are mediated through the TrkB receptor, a thorough understanding of the molecular basis of the function of 7,8-DHF is not yet clear. 7,8-DHF is known to bind to the TrkB extracellular domain in the region of the cysteine cluster 2 (CC2) and the leucine rich region (LRR) [1,5]. Our study suggests that 7,8-DHF may also interact with and additionally bind to the Ig2 domain of the TrkB-D5 extracellular domain. This additional binding site could mediate, at least in part, the 7,8-DHF binding affinity to the TrkB. Our findings are in agreement with previous observations that another TrkB ligand, brain derived neurotrophin factor (BDNF), binding to TrkB is partly mediated through the Ig2 domain in TrkB receptor which contributes to the receptor dimerization [4]. The Ig2 domain possesses an *N*-glycosylation site that could potentially mediate the ligand receptor interaction [8].

The VEGF receptor super-family is another class of tyrosine kinase receptors that play a critical role in the retina. In addition to its involvement in neovascularisation associated with several proliferative disorders, abnormal VEGF expression is implicated in several ocular disease conditions such as macular edema associated with diabetic retinopathy [9], choroidal neovascularisation associated with age-related macular degeneration (AMD) [10], neovascular glaucoma and fibrotic complications of glaucoma filtration surgery *etc*. [11]. VEGFR2 is well expressed in the retina and is believed to predominantly regulate the cellular actions of VEGF [12,13]. Flavonoids have been reported to play a role in the inhibition of VEGFR2 and thus suppress angiogenesis and proliferation of vascular endothelial cells [14,15]. VEGFR2 is thus important target to study the biological effects of 7,8-DHF and other similar flavonoid compounds.

We report here for the first time a dual action of compound 7,8-DHF on TrkB and the VEGFR2 receptor. Using a combination of bioinformatics and biochemical approaches we have provided critical additional insights into the molecular interactions of 7,8-DHF with both the TrkB and the VEGFR2 receptor. Structurally related derivatives of 7,8-DHF are extensively compared to determine the interactions and binding parameters with the TrkB and VEGFR2 receptors. This study also illustrates the effects of 7,8-DHF treatment on the activity of VEGFR2 in both 661W photoreceptor cells as well as in the rat retina.

## 2. Results

### 2.1. Molecular Determinants of 7,8-DHF Binding with TrkB and VEGFR2

**Figure 1:**
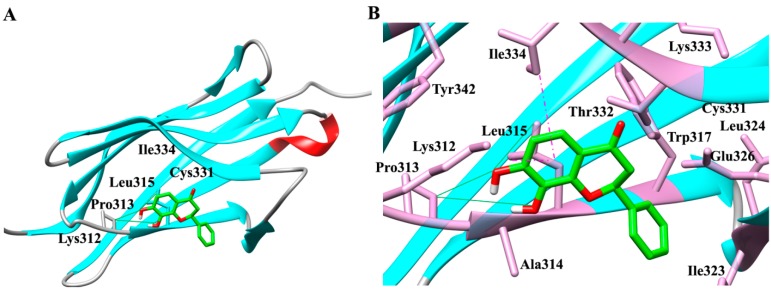

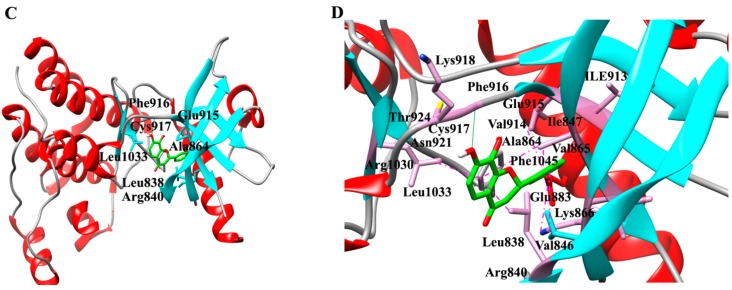
Interaction and binding mode of 7,8-dihydroxyflavone (7,8-DHF) with TrkB and VEGFR2. (**A**) Docking of TrkB (ribbon structure) with the 7,8-DHF (wire-frame) showing critical residues involved in interaction; (**B**) Enlarged view of the interaction pocket within 5.5 Å around the ligand, 7,8-DHF-TrkB complex; (**C**) Docking of VEGFR2 (ribbon structure) with the 7,8-DHF (wire-frame) highlighting important residues involved in interaction; (**D**) Enlarged view of the interaction pocket within 5.5 Å around the ligand, 7,8-DHF-VEGFR2 complex. Red (α-helix), cyan (β-sheets) grey (random coil). Green strong line denotes the hydrogen bonding and pink dashed line reflects *pi*–sigma interactions and stacking. The images were generated with the Discovery Studio 4.0 Client (Accelrys, Inc., San Diego, CA, USA).

The interactions of 7,8-DHF with TrkB and theVEGFR2 receptor were analysed using a molecular docking approach. TrkB-domain5 (TrkB-D5) and VEGFR2 structures were subjected to 7,8-DHF binding *in silico* using AutoDock4.2 to reveal the best binding modes of 7,8-DHF. Our studies revealed that the binding site of TrkB-D5 comprised of Lys^312^, Pro^313^, Ala^314^, Leu^315^, Trp^317^, Ile^323^, Leu^324^, Glu^326^, Cys^331^, Thr^332^, Lys^333^, Ile^334^ and Tyr^342^ residues. Hydrogen bonding with Leu^315^ and Ile^334^ indicated these to be critical residues involved in interaction with 7,8-DHF (Figure 1A,B). In the case of 7,8-DHF docking with VEGFR2, the binding site was selected based on its previously reported interactions with 2-anilino-5-aryl-oxazole (AAX), a VEGFR2 inhibitor (PDB id. 1Y6B). The amino acids Leu^838^, Arg^840^ Ile^847^, Ala^864^, Val^865^, Lys^866^, Glu^883^, Ile^913^, Val^914^, Phe^916^, Cys^917^, Lys^918^, Asn^921^, Thr^924^, Arg^1030^, and Leu^1033^ were observed to comprise the binding site of VEGFR2 protein. AAX extraction and docking of 7,8-DHF showed key hydrogen bond interaction with Cys^917^ residues of VEGFR2 protein (Figure 1C,D).

### 2.2. Binding Interactions of TrkB and VEGFR2 Receptors with 7,8-DHF Derivatives

**Figure 2:**
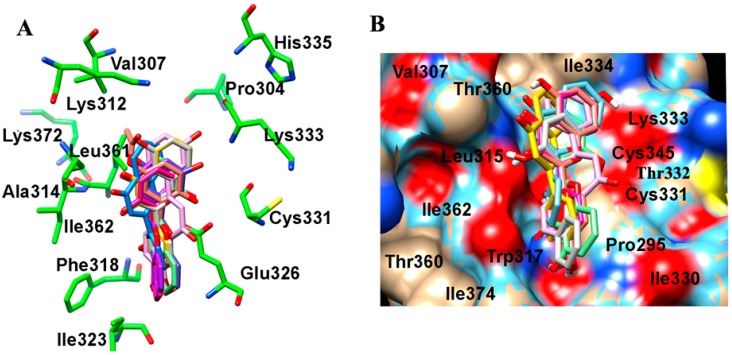
Molecular modelling showing interaction of 7,8-DHF derivatives with the TrkB. (**A**) Amino acid interactions of TrkB (green) with the stick model of 7,8-DHF derivatives (red, blue, yellow, cyan, pink and orange); (**B**) Space filled surface view model of TrkB (color by heteroatom) to depict the binding pocket of 7,8-DHF derivatives (stick model) in TrkB domain.

In order to understand the mechanism of TrkB and VEGFR2 binding with 7,8-DHF, we evaluated various interaction parameters of several structural derivatives of 7,8-DHF (Table 1). A panel of 37 dihydroxy flavonoid derivatives were selected and individually docked to both TrkB-D5 and VEGFR2 using Lamackrian Genetic Algorithms (LGA). The docking scores, predicted binding energies, inhibitory constants and other energies were calculated (Table 2 and Table 3). Among the TrkB–Flavone derivative complex clusters, the lowest binding energy complexes have been listed in Table 2. A binding energy of −5.71 kcal mol^−1^ was observed to be associated with the binding of 7,8-DHF with human TrkB-D5. An inhibitory constant (*K*_i_) of 64.79 µM was calculated for 7,8-DHF-TrkB complex binding, which correlated well with its binding energy. In general, all 37 flavonoid derivatives could be divided into two parts: scaffold I comprised of 2,3-dihydro-4*H*-chromen-4-one, and scaffold II formed by a 2-phenyl group (Table 1). The study showed that 14 of the dihydroxy flavonoid derivatives interacted through scaffold I (2,3-dihydro-4*H*-chromen-4-one), seven dihydroxy flavonoid derivatives through scaffold II (phenyl ring) and the remaining 16 involved interactions through both scaffold I and scaffold II with the TrkB protein (Figure 2A). Scaffold I binding and orientation was largely conserved amongst all the 7,8-DHF derivatives. The scaffold I moiety binding region comprised Lys^312^, Ala^314^, Glu^326^, Thr^332^, and Ile^334^ residues (Figure 2B). In scaffold I, ring A formed a *pi*–alkyl and *pi*–sigma stacking interaction between Leu^315^ and Ile^334^ respectively, while the 7,8-dihydroxy group interacted with the main chain O atom of Pro^313^ and N atom of Leu^315^ respectively through hydrogen bonding.

**Table 1:** Structural parameters of thirty-seven di-hydroxy flavonoid derivatives including 7,8-DHF. 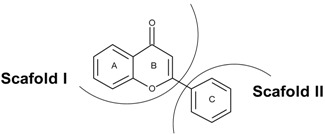

Compounds	Ring A	Ring B	Ring C	Scaffolds
7,8-DHF	7,8 di-OH	–	–	I
C1	–	2,3 di-OH	–	I
C2	–	–	2ʹ,3ʹ di-OH	II
C3	–	–	2ʹ,5ʹ di-OH	II
C4	5-OH	–	2ʹ-OH	I and II
C5	–	–	2ʹ,5ʹ di-OH, 5ʹ-acetate	II
C6	5-OH	–	4ʹ-OH	I and II
C7	–	3-OH	2ʹ-OH	I and II
C8	–	3-OH	4ʹ-OH	I and II
C9	–	–	3ʹ,4ʹ di-OH	II
C10	–	–	3ʹ,4ʹ di-OH, 4ʹ-glucoside	II
C11	5-OH, 6,7 di-methoxy	3-methoxy	3ʹ-OH, 4ʹ-methoxy	I and II
C12	3,5 di-OH	–	–	I
C13	–	–	3ʹ,5ʹ di-OH	II
C14	3,6 di-OH	–	–	I
C15	3,7 di-OH	–	–	I
C16	6-OH	–	4ʹ-OH	I and II
C17	7-OH	–	4ʹ-OH	I and II
C18	5-OH	–	3ʹ-OH	I and II
C19	5-OH, 7-methoxy	–	4ʹ-OH, 3ʹ-methoxy	I and II
C20	5,6 di-OH	–	–	I
C21	5,7 di-OH	–	4ʹ-methoxy	I and II
C22	5,7 di-OH	–	–	I
C23	5,7 di-OH	–	7-benzoate	I and II
C24	5,7 di-OH, 7 β-monoglucoside	–	–	I
C25	5,8 di-OH	–	–	I
C26	6,7 di-OH	–	–	I
C27	6,8 di-Cl	–	3ʹ,5ʹ di-OH	I and II
C28	5-OH, 6-methoxy, 7-*O*-glucoside	–	4ʹ-OH	I and II
C29	7-OH	–	2ʹ-OH	I and II
C30	7-OH, 7-glucoside	–	2ʹ-OH	I and II
C31	7-OH	–	3ʹ-OH	I and II
C32	7-OH, 7-glucoside	–	4ʹ-OH	I and II
C33	7-OH, 7-rutinoside	–	4ʹ-OH	I and II
C34	5, 6-OH, 7-d-glucuronic acid	–	–	I
C35	8-OH	–	2ʹ-OH	I and II
C36	7-OH, 8-β-d-glucopyranosyl	–	4ʹ-OH	I and II

**Table 2:** 7,8-DHF and 36 di-hydroxy flavonoid derivatives with corresponding energies obtained from docking with TrkB-D5 using AutoDock program. *BE^e^* Estimated binding free energy in kcal mol^−1^; *K*_i_ Inhibitory constant in micro-molar; *IME^e^* Final Intermolecular Energy in kcal mol^−1^; *V_dw_–H_b_–D_s_* van der Waals-hydrogen bond-desolvation energy component of binding free energy in kcal mol^−1^; *E^e^* Electrostatic energy in kcal mol^−1^; *IE^e^* Final total internal energy in kcal mol^−1^; *TFE^e^* Torsional free energy in kcal mol^−1^.

Compound Name	*BE^e^* (kcal/mol)	*K*_i_ (µM)	*IME^e^* (kcal/mol)	*V_dw_–H_b_–D_s_* (kcal/mol)	*E^e^* (kcal/mol)	*IE^e^* (kcal/mol)	*TFE^e^* (kcal/mol)
7,8-DHF	−5.71	64.79	−5.84	−5.6	−0.24	−0.69	0.82
C1	−5.94	44.35	−5.97	−5.91	−0.06	−0.8	0.82
C2	−5.91	46.34	−6.57	−6.12	−0.45	−0.16	0.82
C3	−5.28	133.89	−5.54	−5.34	−0.2	−0.57	0.82
C4	−6.13	32.06	−6.28	−5.94	−0.34	−0.67	0.82
C5	−5.96	43.12	−6.84	−6.41	−0.43	−0.21	1.10
C6	−5.65	71.59	−5.77	−5.63	−0.13	−0.71	0.82
C7	−5.63	75.12	−6.09	−5.71	−0.38	−0.36	0.82
C8	−5.52	89.37	−5.95	−5.85	−0.09	−0.4	0.82
C9	−6.02	38.62	−6.7	−6.51	−0.19	−0.14	0.82
C10	−7.10	6.21	−6.33	−6.08	−0.25	−3.24	2.47
C11	−7.42	3.62	−7.67	−7.49	−0.17	−1.68	1.92
C12	−6.43	19.41	−6.36	−6.01	−0.35	−0.89	0.82
C13	−5.18	158.66	−5.85	−5.6	−0.25	−0.15	0.82
C14	−5.24	143.46	−5.51	−5.37	−0.14	−0.56	0.82
C15	−5.36	118.3	−5.86	−5.58	−0.28	−0.32	0.82
C16	−5.28	135.23	−5.94	−5.76	−0.18	−0.16	0.82
C17	−5.49	94.66	−6.14	−5.97	−0.18	−0.17	0.82
C18	−5.81	55.47	−5.95	−5.67	−0.28	−0.68	0.82
C19	−6.17	30.1	−6.37	−5.99	−0.38	−1.17	1.37
C20	−6.00	39.92	−5.81	−5.52	−0.29	−1.01	0.82
C21	−5.97	42.38	−6.30	−6.11	−0.19	−0.76	1.10
C22	−5.46	98.9	−5.56	−5.36	−0.20	−0.73	0.82
C23	−6.47	18.12	−6.68	−6.51	−0.17	−1.16	1.37
C24	−6.85	9.56	−7.02	−6.88	−0.13	−2.3	2.47
C25	−6.08	35.18	−5.82	−5.78	−0.04	−1.08	0.82
C27	−6.29	24.48	−6.92	−6.72	−0.2	−0.19	0.82
C28	−7.90	1.61	−8.31	−8.05	−0.27	−2.61	3.02
C29	−5.41	108.5	−6.05	−5.76	−0.29	−0.18	0.82
C30	−6.53	16.21	−7.06	−6.84	−0.22	−1.94	2.47
C31	−5.62	76.09	−6.29	−5.91	−0.38	−0.15	0.82
C32	−5.89	47.88	−6.25	−5.99	−0.26	−2.11	2.47
C33	−6.16	4.82	−4.05	−3.86	−0.19	−2.68	3.57
C34	−7.50	3.18	−6.97	−6.38	−0.6	−3.27	2.74
C35	−5.64	72.83	−5.68	−5.26	−0.42	−0.79	0.82
C36	−6.77	10.99	−7.78	−7.15	−0.63	−1.45	2.47

**Figure 3:**
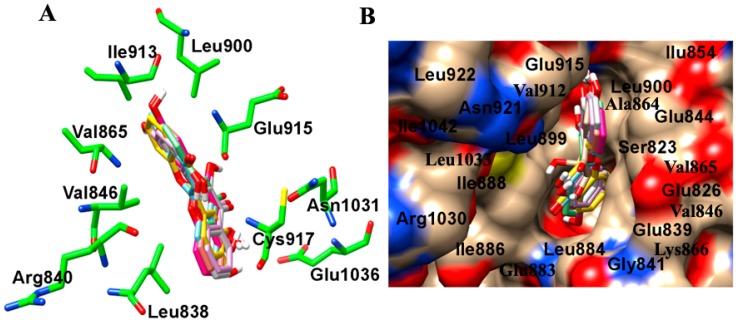
Molecular modelling showing interaction of 7,8-DHF derivatives with the VEGFR2. (**A**) Amino acid interactions of VEGFR2 (green) with the stick model of 7,8-DHF derivatives (red, blue, yellow, cyan, pink and orange); (**B**) Space filled surface view model to depict the binding pocket of 7,8-DHF derivatives (stick model) in VEGFR2 (color by heteroatom).

Further, we investigated the interactions of these flavone derivatives with VEGFR2. The study indicated interactions of 18 dihydroxy flavonoids by scaffold I, seven dihydroxy flavonoids through scaffold II, and the remaining 12 dihydroxy flavonoid derivatives through both the scaffold I and scaffold II with VEGFR2 (Table 3). The binding pocket of VEGFR2 was comprised of Val^846^, Ala^864^, Val^865^, Lys^866^, Glu^883^, Val^914^, Glu^915^, Phe^916^, and Leu^1033^ residues (Figure 3A,B). In scaffold I, ring A formed a *pi*–alkyl and *pi*–sigma stacking interaction with Ala^864^ and Leu^1033^ respectively. Scaffold II also showed 3 *pi*–alkyl interactions with Val^846^, Ala^864^, Lys^866^ and one *pi*–sigma stacking interaction with Val^914^. The 7,8-dihydroxy group interacted with the carboxyl O and amino N atom of Cys^917^ and amino N atom of Leu^315^ through hydrogen bonding. Scaffold I binding and orientation was approximately conserved amongst all the 7,8-DHF derivatives (Figure 2 and Figure 3). With respect to 7,8-DHF interaction, a binding score of −7.76 kcal mol^−1^ and *K*_i_ of 2.04 µM were observed when compared to that calculated for AAX, a known VEGFR2 inhibitor with binding score of −9.68 kcal mol^−1^ and *K*_i_ 0.08 µM (Table 3). Potential carcinogenicity and mutagenicity of various dihydroxyflavone derivatives in cells and rodents was predicted using the ToxPredict tool [16] (Table S1). ToxPredict studies demonstrated 7,8-DHF to be a non-carcinogenic and non-mutagenic flavonoid with minimal toxicity potential compared to all other derivatives.

**Table 3:** 7,8-DHF and 36 di-hydroxy flavonoid derivatives with corresponding energies obtained from redocking validation followed by docking with VEGFR2 protein using AutoDock program. *BE^e^* Estimated binding free energy in kcal mol^−1^; *K*_i_ Inhibitory constant in micro-molar; *IME^e^* Final Intermolecular Energy in kcal mol^−1^; *V_dw_–H_b_–D_s_* van der Waals-hydrogen bond-desolvation energy component of binding free energy in kcal mol^−1^; *E^e^* Electrostatic energy in kcal mol^−1^; *IE^e^* Final total internal energy in kcal mol^−1^; *TFE^e^* Torsional free energy in kcal mol^−1^.

Compound Name	*BE^e^* (kcal/mol)	*K*_i_ (µM)	*IME^e^* (kcal/mol)	*V_dw_-H_b_-D_s_* (kcal/mol)	*E^e^* (kcal/mol)	*IE^e^* (kcal/mol)	*TFE^e^* (kcal/mol)
AAX	−9.68	0.08	−10.43	−10.42	−0.01	−1.44	+2.20
7,8-DHF	−7.76	2.04	−8.09	−7.97	−0.12	−0.50	+0.82
C1	−6.69	12.49	−6.49	−6.49	+0.00	−1.03	+0.82
C2	−7.01	7.29	−7.28	−7.18	−0.10	−0.56	+0.82
C3	−6.66	13.11	−6.91	−6.86	−0.05	−0.57	+0.82
C4	−6.97	7.72	−7.17	−7.09	−0.08	−0.62	+0.82
C5	−7.44	3.51	−8.27	−8.08	−0.19	−0.27	+1.10
C6	−7.27	4.66	−7.39	−7.23	−0.15	−0.71	+0.82
C7	−7.08	6.44	−7.09	−6.97	−0.11	−0.82	+0.82
C8	−7.19	5.41	−7.52	−7.25	−0.27	−0.49	+0.82
C9	−7.07	6.52	−7.76	−7.59	−0.17	−0.14	+0.82
C10	−9.41	0.13	−9.64	−9.20	−0.44	−2.24	+2.47
C11	−6.33	22.82	−6.69	−6.49	−0.20	−1.56	+1.92
C12	−7.41	3.71	−7.16	−7.13	−0.03	−1.07	+0.82
C13	−7.25	4.84	−7.91	−7.58	−0.33	−0.17	+0.82
C14	−7.31	4.41	−7.61	−7.48	−0.14	−0.52	+0.82
C15	−7.30	4.46	−7.69	−7.56	−0.13	−0.44	+0.82
C16	−7.27	4.69	−7.93	−7.68	−0.25	−0.17	+0.82
C17	−7.54	2.99	−8.19	−7.89	−0.30	−0.17	+0.82
C18	−7.08	6.48	−7.19	−6.88	−0.31	−0.71	+0.82
C19	−7.73	2.16	−7.92	−7.79	−0.13	−1.18	+1.37
C20	−7.75	2.10	−7.87	−7.73	−0.14	−0.70	+0.82
C21	−7.23	5.03	−7.56	−7.48	−0.08	−0.77	+1.10
C22	−7.53	3.02	−7.61	−7.55	−0.06	−0.74	+0.82
C23	−8.34	0.77	−8.61	−8.56	−0.05	−1.10	+1.37
C24	−8.17	1.03	−8.47	−8.23	−0.24	−2.17	+2.47
C25	−7.17	5.54	−7.04	−6.99	−0.05	−0.95	+0.82
C26	−7.60	2.68	−7.77	−7.58	−0.19	−0.65	+0.82
C27	−7.50	3.17	−8.14	−7.85	−0.29	−0.19	+0.82
C28	−8.76	0.38	−9.01	−8.76	−0.25	−2.77	+3.02
C29	−6.93	8.28	−7.16	−7.12	−0.04	−0.59	+0.82
C30	−7.96	1.47	−8.23	−7.81	−0.42	−2.20	+2.47
C31	−7.46	3.40	−8.11	−7.65	−0.47	−0.17	+0.82
C32	−8.43	0.66	−9.03	−8.68	−0.36	−1.86	+2.47
C33	−8.14	1.09	−7.83	−7.65	−0.18	−3.88	+3.57
C34	−9.22	0.174	−9.45	−8.47	−0.98	−2.52	+2.74
C35	−6.73	11.57	−6.60	−6.56	−0.04	−0.96	+0.82
C36	−8.29	0.84	−8.21	−8.07	−0.14	−2.55	+2.47

### 2.3. Molecular Dynamics (MD) of 7,8-DHF-TrkB and 7,8-DHF-VEGFR2 Complex

**Figure 4:**
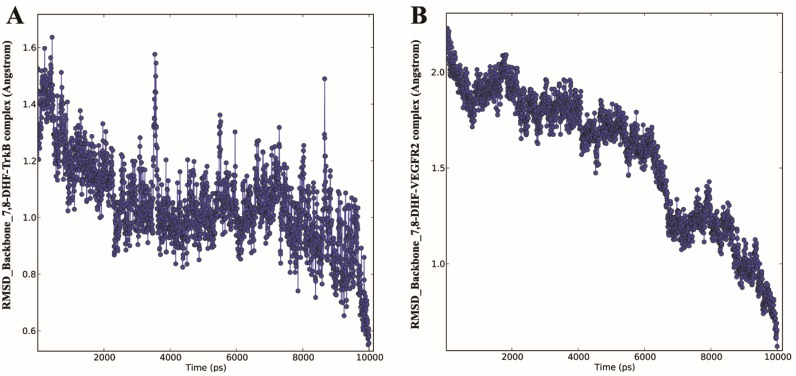
The MD simulation time *vs.* RMSD of the backbone atoms. (**A**) 7,8-DHF-TrkB protein complex; (**B**) 7,8-DHF-VEGFR2 protein complex.

**Figure 5:**
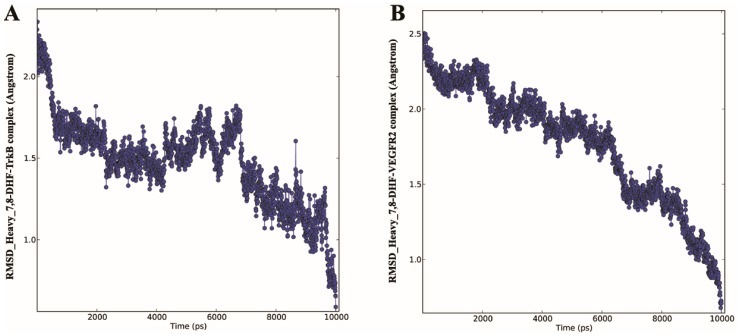
MD simulation time *vs.* RMSD of the heavy atoms. (**A**) 7,8-DHF-TrkB protein complex; (**B**) 7,8-DHF-VEGFR2 protein complex.

MD simulation provides information about the internal motions of the receptor–ligand complex treated in a flexible condition in the solvent with respect to time. In order to confirm the binding mode of 7,8-DHF-TrkB-D5 and 7,8-DHF-VEGFR2 docking complexes, MD simulation was performed using the Desmond program 3.2 [17]. MD simulations were carried out in an *in silico* environment mimicking physiological condition of pH and molarity. The dynamic properties of 7,8-DHF-TrkB-D5 and 7,8-DHF-VEGFR2 docking complexes were analysed using trajectory data obtained from 10 ns MD simulations indicating effective receptor–ligand binding under the above conditions. The trajectory of 7,8-DHF with TrkB-D5 and VEGFR2 docking complexes were plotted for root mean square fluctuation (RMSF) (Figure S1), energy (Figure S2) and root mean square deviation (RMSD) (Figure 4 and Figure 5).

RMSD plot for backbone and heavy atoms (Figure 4 and Figure 5) indicated a subtle rearrangement in the initial conformation of the docking complex that eventually stabilised following molecular simulation. The overall range of RMSD of 7,8-DHF-TrkB-D5 and 7,8-DHF-VEGFR2 complex was 0.3–1.7 and 0.2–2.3 Å for backbone atoms, respectively (Figure 4A,B). For heavy atoms the average RMSD for the 7,8-DHF-TrkB-D5 and 7,8-DHF-VEGFR2 complex was observed to be 0.2–2.8 and 0.2–2.5 Å respectively (Figure 5A,B). The RMSF of the residues were approximated by averaging all the atoms of the given protein. RMSF analysis indicated that all backbone (blue) and most of the side chain residues (red) were within the acceptable limit of 2.5 Å. Fluctuations for some of the side-chain residues for TrkB complex exceeded 2.5 Å but was below 3.0 Å (Figure S1A). Similar patterns of RMSF was evident with respect to VEGFR2, where most of the backbone (blue) and side chain (red) residues were within the limit of 2.5 Å (Figure S1B). The lower atomic fluctuation for active site residues reflected small conformational changes. The energy, RMSD and RMSF plots illustrated that the 7,8-DHF-TrkB-D5 and 7,8-DHF-VEGFR2 docking complex were observed to be stable throughout MD simulation. 7,8-DHF-TrkB-D5 and 7,8-DHF-VEGFR2 molecular interactions were also monitored to assess the structural flexibility of the docked complex (Figure S3). Molecular analysis of 7,8-DHF complex with TrkB-D5 showed 18 (O) and 20 (O) atoms of 7,8-DHF to be involved in hydrogen bonding with 250 (N) of Leu^315^ and 236 chiral (C) atom of Lys^312^ respectively (Figure S3A). The trajectory analysis of MD simulation further showed hydrogen bond formation between atom 19 H11 of 7,8-DHF and atom 241 (O) of Pro^313^ as well as atom 21 (H) of 7,8-DHF and atom 241 (O) Pro^313^. In addition *pi*–sigma interaction was also observed between the B ring of 7,8-DHF and atom 265 (C) of Leu^315^. Hydrogen bonding and *pi*–sigma bonding with the residues Pro^313^ and Leu^315^ in MD simulation may indicate a potential conformational change in TrkB (Figure 1B and Figure S3A). In VEGFR2 complex simulation, atom 20 (O) of 7,8-DHF was observed to form hydrogen bonds with atom 743 (N) of Cys^917^ and atom 746 (O) of Cys^917^ (Figure S3B). The trajectory analysis of MD simulation showed hydrogen bonding between atom 19 H11 of 7,8-DHF and atom 726 (O) of Glu^915^ as well as atom 18 (O) of 7,8-DHF and atom 733 (H) Phe^916^ which was additional to that of Cys^917^ hydrogen bond interaction observed in molecular docking analysis (Figure 1D and Figure S3B).

### 2.4. 7,8-DHF Treatment Leads to Loss of VEGFR2 Activity

**Figure 6:**
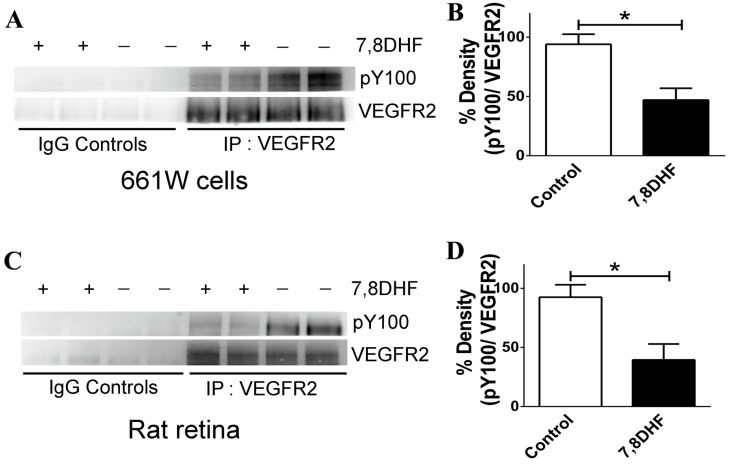
7,8-DHF exerts an inhibitory effect on the VEGFR2. (**A**) Immunoprecipitation of VEGFR2 was carried out from 661W cell lysates followed by probing with the VEGFR2 and PY100 antibodies; (**B**) Quantification illustrated a decline in VEGFR2 phosphorylation upon treatment with 7,8-DHF (* *p* < 0.04); (**C**) Immunoprecipitation of VEGFR2 from retinal lysates followed by probing with the VEGFR2 and PY100 antibodies; (**D**) Quantification illustrated a decline in VEGFR2 phosphorylation from rats treated with 7,8-DHF (* *p* < 0.05).

7,8-DHF is known to bring about substantial activation of the TrkB receptor both *in vitro* and *in vivo* [18]. Here we investigated whether 7,8-DHF has any effect on the activation of the VEGFR2 receptor. The VEGFR2 was immunoprecipitated from the 661W cell lysates and the blots probed with the pY100 antibody to detect the changes in phosphorylation status of the VEGFR2 receptor. Contrary to that, observed in the case of 7,8-DHF effects on TrkB receptor, we observed a dephosphorylation of the VEGFR2 in the cells that were pre-treated with the flavonoid derivative. Quantification of the band intensities showed a significant loss of the VEGFR2 activity (*p* < 0.04) (Figure 6A,B). The effects of the drug on VEGFR2 *in vivo* were investigated by immunoprecipitating VEGFR2 from the rat retinal lysates. Samples from animals treated with 7,8-DHF demonstrated a loss of VEGFR2 phosphorylation using the pY100 antibodies compared to the control retinal samples (*p* < 0.05) (Figure 6C,D). Non-immune IgGs were used as control for immunoprecipitations. Band intensities were normalised to the total amount of VEGFR2 immunoprecipitated in each case to ensure that phosphorylation changes were not attributed to differences in amounts of total immunoprecipitated protein.

## 3. Discussion

This study investigated the molecular interactions underlying 7,8-DHF and various other dihydroxyflavone derivatives with the TrkB receptor using a combination of molecular docking and dynamics studies. We also examined for first time molecular interactions between various flavonoid derivatives including 7,8-DHF with the VEGFR2. 7,8-DHF is an agonist of TrkB receptor and its treatment leads to upregulation in the tyrosine phosphorylation on TrkB residues and activate its downstream signalling [19]. Intriguingly, a combination of molecular modelling and biochemical approaches has revealed that 7,8-DHF could act as an inhibitor of the VEGFR2. This suggestion corresponded with the previous observations that flavonoids inhibit the VEGFR2 activity in human umbilical vein endothelial cells [20]. The inhibitory constant of 7,8-DHF for VEGFR2 was calculated and found to be 2.04 µM indicating a ~32 fold higher inhibitory constant for the VEGFR2 as compared to a theoretical value of *K*_i_ 64.79 µM for TrkB. The high *in silico* *K*_i_ suggested that 7,8-DHF did not have a significant inhibitory effect on TrkB [1,21] as compared to that observed in the case of VEGFR2 [22,23].

The *in silico* docking approaches based on topological surface geometry complementarities for 7,8-DHF-TrkB and 7,8-DHF-VEGFR2 complexes indicated formation of hydrogen bond networks between surface amino acid residues [24]. The stable behaviour of both the complexes could be attributed to van der Waals forces and atomic contact energies [25]. Molecular docking of 7,8-DHF with TrkB showed presence of 3 H-bonds between 7,8-DHF and TrkB protein at the Ig2 domain of extracellular region [8]. Interestingly, these interactions are in addition to already known interactions of 7,8-DHF with the cysteine cluster 2 (CC2) region of TrkB which is formed by the disulfide linkage of Cys145–Cys121 and Cys123–Cys163 residues and the leucine rich region (LRR) [8]. Using a truncated binding assay, Jang *et al.* showed that 7,8-DHF strongly associated with the CC2 domain and also partially interacted with the leucine-rich motif domain. This additional binding site at Ig2 may play a role in stabilising or further enhancing the 7,8-DHF binding to the TrkB. Similar involvement of the TrkB Ig2 domain in interactions with BDNF were observed with potential contributions to the TrkB receptor dimerization [4]. The *N*-glycosylation site in the Ig2 domain could also play a role in ligand receptor interaction, but further studies are required to establish this [8].

Molecular simulations revealed the presence of additional H-bonds increasing the total number to five, and also indicated formation of *pi*–sigma bonds. The 7,8-DHF-VEGFR2 complex also showed formation of two hydrogen bonds with Cys^917^ after molecular docking. Molecular simulations further showed additional 5 H-bonds involving Glu^915^, Phe^916^ and Cys^917^ residues. Detection of additional bonds and interactions following molecular simulations indicated that the protein-ligand complexes acclimatize and achieve a more stable configuration over a period of time. No significant changes were observed in the total energy of either of the protein-ligand complexes within the 10ns simulation period indicating that the complexes attained a stable conformation (Figure S2A,B).

The differential effects of 7,8-DHF on TrkB and VEGFR2 were expected as the two membrane receptors belong to two independent superfamilies of receptor tyrosine kinases. VEGFR2 in addition to its several other unique structural features does not possess either the CC2 or LRR domains in its extracellular region. Further, the VEGFR2 ATP binding site has Val^914^, Phe^916^, and Cys^917^ residues which are critically involved in the hydrogen bond and *pi*–sigma interactions with the ligand; these residues are not present in TrkB. These residues are also absent in other kinases such as insulin-like growth factor 1 receptor (IGF1R), serine/threonine-protein kinase 4 (STK4), phosphatidylinositol 4,5-bisphosphate 3-kinase (PK3CG) and CUB domain-containing protein 1 (CDCP1) (Figure S4A). Further, tertiary structural analysis revealed that Val^568^ (TrkB) which corresponds to Val^846^ (VEGFR2) is buried in the TrkB structure and is not surface accessible for development of bond formation. These observations suggested that the absence of these structural motifs in TrkB could be the reason underlying exclusion of 7,8-DHF interactions with the ATP binding site of TrkB in contrast to that observed in VEGFR2 (Figure S4). Together these findings suggest that the inhibitory effects observed in VEGFR2 are not generic in nature and may not be generalised across different kinase families.

The relevance of the predictive value of our *in silico* studies was investigated in the photoreceptor 661W cells in culture as well as in the rat retina under *in vivo* conditions. 7,8-DHF treatment has been shown to activate the TrkB signalling and reduce apoptosis by activating the downstream processes of Akt and Erk1/2 pathways. We chose 661W cells as these have been shown to express VEGFR2 [26]. Our previous studies have established that 7,8-DHF treatment can activate the TrkB and its downstream signalling in the retinal ganglion cells as well as in RGC-5 cells. VEGFR2 is known to undergo tyrosine (Tyr) autophosphorylation at residues 951/996 and 1054/1059 in response to ligand binding, and undergo activation [27]. Phosphorylation leads to rapid recruitment of intracellular adapter proteins which is essential process to execute the VEGF stimulated signalling as well as mediate survival of endothelial cells and regulate angiogenesis process [28,29,30]. In order to evaluate whether 7,8-DHF treatment had any effect on the activity of the VEGFR2, we evaluated changes in the Tyr phosphorylation of VEGFR2 in both the 661W cells in culture as well as in the rat retinal tissue. Immunoprecipitation of VEGFR2 followed by probing the blots with pY100 antibody demonstrated that Tyr phosphorylation was significantly reduced upon treatment with 7,8-DHF. The experiments were conducted on immunoprecipitated proteins using specific VEGFR2 antibodies and not whole lysates to eliminate possible interfering signals from other proteins. Appropriate controls were maintained in the form of non-immune IgGs for both the control as well as 7,8-DHF treated samples. This experiment established that 7,8-DHF has a dual effect in suppressing the VEGFR2 actions by reducing its activity in addition to its known agonistic effects on TrkB. These experimental observations corroborate our *in silico* predictions. Briefly, the fact that similar inhibitory effect was observed in the rat retinal tissues upon 7,8-DHF treatment validate our 661W results and reassure that the inhibitory effects are not an experimental artefact or not a cell specific phenomenon. The loss in VEGFR2 activity can potentially be attributed to 7,8-DHF interactions with key active site residues Glu^915^, Phe^916^, and Cys^917^ that may give rise to conformational changes in the geometry of the protein as observed in the molecular dynamic studies (Figure 4 and Figure 5).

Concurrent effects of a TrkB agonist as an inhibitor of VEGFR2 actions could have great application in the development of innovative therapeutics. In wet AMD for example, 7,8-DHF may enhance TrkB signalling and promote critical neuroprotective pathways while simultaneously downregulating VEGFR2, thereby inhibiting unregulated neovascularisation in the retina. The data also suggested that treatment with 7,8-DHF may have clinical importance in other retinal vascular diseases including diabetic retinopathy and associated macular edema in retinal vein occlusions, based on its ability to activate TrkB and inhibit VEGFR2 receptors at the same time. Since many patients have co-existing pathologies of glaucoma, AMD and diabetes, an agent with these properties could provide additional benefits.

## 4. Experimental Section

### 4.1. Chemicals

7,8-Dihydroxyflavone was purchased from Tocris Bioscience, Bristol, UK. Anti-BDNF (sc-546) and anti-TrkB (sc20542) antibodies were obtained from Santa Cruz Biotechnology (Santa Cruz, CA, USA). Anti-VEGF antibody (Abcam, Melbourne, VIC, Australia; ab46154) and VEGFR2 antibody (Cell Signaling Technology, Danvers, MA, USA; 55B11) were also used for western blotting. β-Actin antibody was obtained from Sigma, USA. All other reagents were of analytical grade from Sigma (St. Louis, MO, USA).

### 4.2. Animal Experiments

Animal experiments were conducted in accordance with the Australian code of practice for the care and use of animals for scientific purposes and the guidelines of the Association for Research in Vision and Ophthalmology (ARVO) statement for the use of animals in ophthalmic and vision research and were approved by the Macquarie University Animal Ethics Committee (2012/31), NSW, Australia. Male Sprague-Dawley rats were obtained from Animal Research Centre, Perth, Australia and maintained in the animal house in cyclic light (12 h on; 12 h off; ~300 lux), in an air-conditioned room with controlled temperature (21 ± 2 °C) and with free access to water and rodent chow.

### 4.3. Selection and Preparation of Macromolecule

Crystal structure of the TrkB-D5 domain bound to Neurotrophin-4/5 (PDB id: 1HCF) [31] and VEGFR2 protein (PDB id: 1Y6B) [32] from human was retrieved from a protein databank [33]. TrkB-D5 domain contains four chains A, B, X and Y. The A and B chains constitute Neurotrophin 4, which forms homodimer. The chains X and Y form BDNF/ NT-3 protein. Only chain X of PDB id 1HCF was considered for these studies. On the other hand, VEGFR2 contains only one chain A bound to 2-anilino-5-aryl-oxazole inhibitor. The selection of two proteins was carried out on the basis of resolution and organism from which derived. Resolution for TrkB-D5 and VEGFR2 was 2.70 Å and 2.10 Å respectively. The optimization of proteins was carried out using UCSF Chimera software (Pettersen *et al.*, San Francisco, CA, USA), implying amber parameters, followed by minimization with MMTK method in 500 steps with a step size of 0.02 Å [34]. The active site residues of the binding pocket were determined from the Castp server [35] for the TrkB-D5 domain, and bound ligand in case of VEGFR2.

### 4.4. Selection and Preparation of Dihydroxy Flavones Derivatives

The three-dimensional (3D) structures of dihydroxy flavones derivatives were collected from the pubchem database [36]. In total, 37 derivatives were collected including 7,8-dihydroxy flavones, 7,8-DHF (Table 1) and was built using ChemDraw Ultra 8.0 (Cambridgesoft, Waltham, MA, USA). The energy minimization was performed using the Austin Model-1 (AM1) [37] until the root mean square (RMS) gradient value became smaller than 0.100 kcal/mol Å and then molecules were subjected to re-optimization via MOPAC (Molecular Orbital Package) method [38] until the RMS gradient attained a value lesser than 0.0001 kcal/mol Å using MOPAC.

### 4.5. Molecular Docking

The docking of the 37 dihydroxy derivatives to the binding site of TrkB-D5 and VEGFR2 was performed using the AutoDock 4.2 [39] (The Scripps Research Institute, La Jolla, CA, USA). In order to compare the results from docking protocols, water molecules and ligand (2-anilino-5-aryl-oxazole) were excluded for better docking score. The rotatable bonds of the ligands were set to be free and the protein was treated as a rigid body [37]. Crystal structure of the TrkB-D5 and VEGFR2 protein (1HCF and 1Y6B) was retrieved from the protein databank (http://www.pdb.org/). Rigid docking was performed for studying protein–ligand interactions through AutoDock tools. The atom types and bond types were assigned [40,41]. The polar hydrogen atoms of the enzymes were added, the non-polar hydrogen atoms were merged, Gasteiger charges were assigned and solvation parameters were added. For all ligands, including 7,8-DHF, the non-polar hydrogen atoms were merged, and the Gasteiger charges were assigned. The auxiliary program AutoGrid generated the grid maps. The grid box dimensions were 60 × 60 × 60 Å and 52 × 46 × 56 Å around the active site and the grid spacing was set to 0.375 Å for TrkB-D5 and VEGFR2 protein respectively. The starting positions of all ligands were outside the grid box (>20 Å away from the centre of the binding pocket). Docking was performed using the empirical free energy function together with the LGA [42]. The LGA protocol applied a population size of 150, while 250,000 energy evaluations were used for the 20 LGA runs. In addition, the maximum number of evaluations was set to 27,000; the mutation rate to 0.02; the crossover rate to 0.8; and the elitism rate to 1.0. Estimated inhibition constants (*K*_i_) were used for determination of binding energies of different docking conformations, ranking in accordance to their binding scores [39]. The calculated properties of *K*_i_, binding free energy, electrostatic energy, van der Waals, hydrogen bond, desolvation energy, total intermolecular and torsional energy for 37 DHF derivatives are given in Table 3 for TrkB-D5 and VEGFR2 respectively. Chimera [34], Discovery Studio (DS) Visualizer2.5 (Biovia, San Diego, CA, USA) [43] and LigPlot^+^ software (Roman Laskowski, Hinxton, Cambridge, UK) [44] were used for visualisation and calculation of protein–ligand interactions.

### 4.6. Molecular Dynamics Simulations

MD simulations were performed for the complex of 7,8-DHF-TrkB and 7,8-DHF-VEGFR2 using Desmond 3.2 software (Shaw Research, New York, NY, USA) [45], incorporating OPLS_2005 force field for 10,000 ps (picoseconds) simulation time. The salvation system was maintained in a 100 × 100 × 100 Å orthorhombic box with periodic boundary conditions by adding SPC (forcefield) water molecules [46] for both the complexes. The whole system was neutralized by adding counter ions Na^+^ and Cl^−^ to balance the net charge of the system. In Desmond, equilibration of the whole system was carried out using default protocol made up of a series of restrained minimizations and MD simulations. During simulation, initial coordinates of the protein molecules were slowly relaxed without deviation. The minimized system was relaxed with NPT (number of atom, pressure, and temperature) ensemble restraining non-hydrogen solute atom for 10 ns simulation time. The full system was composed of 17,346 atoms for TrkB-D5 and 36,979 for VEGFR2 complex respectively. The temperature was maintained at 300 K and pressure at 1.01325 bars. Long-range electrostatic interactions were computed using particle-mesh Ewald method [47,48] and van der Waals (VDW) cut-off was set to 9 Å. The SHAKE algorithm was used to satisfy the hydrogen bond geometry constraints during simulation [46]. The full system was simulated to analyse the stability of the 7,8-DHF-TrkB-D5 and 7,8-DHF-VEGFR2 complexes. The dynamic behaviour and structural changes of the complex were analysed by calculating the RMSD and energy fluctuation. The root mean square fluctuations (RMSF) for the backbone and side chain of each residue of TrkB-D5 and VEGFR2 protein were analysed. The 7,8-DHF-TrkB-D5 and 7,8-DHF-VEGFR2 complexes were analysed and monitored for the stability in hydrogen bond interactions.

### 4.7. Cell Culture and Treatment Regimens

Photoreceptor derived 661W cells were maintained in DMEM culture media containing 10% fetal bovine serum and 1% penicillin/streptomycin at 37 °C at 5% CO_2_. Approximately, 2.0 × 10^5^ cells were seeded in each culture dish 6–12 h before treatment [49,50]. Cells were treated with 7,8-DHF (100 nM) and allowed to grow for a period of 24 h before harvesting. For *in vivo* experiments, 7,8-DHF (2 mg/kg) was administered intraperitoneally to the rats. The rat retinas were harvested, flash frozen and sonicated in the lysis buffer for further analysis.

### 4.8. Western Blot and Immunoprecipitations

661W cells and retinal tissues were lysed in lysis buffer (20 mM HEPES, pH 7.4, 1% Triton X-100, 1 mM EDTA) containing (10 μg/mL aprotinin, 10 μM leupeptin, 1 mM PMSF) and (1 mM NaVO_3_, 100 mM NaF, 1 mM Na_2_MoO_4_, 10 mM Na4P_2_O_7_). The proteins were separated by 10% SDS-PAGE and transferred to PVDF membranes as explained previously [51]. The blots were washed three times for 5 min with TTBS (20 mM Tris–HCl (pH 7.4), 100 mM NaCl, and 0.1% Tween 20) and blocked with 5% non-fat dry milk (Bio-Rad Laboratories, Inc., Hercules, CA, USA) in TTBS buffer for 1 h at room temperature. Following primary antibody incubations, immunoblots were incubated with horseradish peroxidase (HRP)-linked secondary antibodies and after extensive washing, antibody detection was accomplished with Supersignal West Pico Chemiluminescent substrate (Pierce Biotechnology Inc., Rockford, IL, USA). Signals were detected using an automated luminescent image analyzer (ImageQuant LAS 4000, GE Healthcare, Pittsburgh, PA, USA). Band intensities were quantified using ImageJ software (NIH, Bethesda, MD, USA). Immunoprecipitation was carried out according to the method described earlier [52] and immunoprecipitates subjected to immunoblot analysis with indicated antibodies in the respective figures. Band intensities were normalized to the total amount of protein immunoprecipitated in each case, and quantified using ImageJ software (NIH, Bethesda, MD, USA).

### 4.9. Statistical Analysis

Data were analysed and graphed using GraphPad Prism software (GraphPad Software, La Jolla, CA, USA). All values with error bars are presented as mean ± SD from given n sizes and compared by Student’s *t-*test for unpaired data. The significance was set at *p* < 0.05.

## 5. Conclusions

Inhibition of VEGF/VEGFR signalling may be critical in several disorders involving unregulated angiogenesis. A poorly monitored treatment, on the other hand, may give rise to unwarranted complications. In the context of retina for example, excess anti-VEGF treatment may cause onset of dry AMD leading to gradual neurodegeneration. In contrast, activation of the neurotrophic factor signalling such as TrkB may play a critical role in protecting against several neurodegenerative disorders including retinal disorders. In this study, we have evaluated the potential of flavonoid derivatives to act as VEGFR2 inhibitors and the same time evaluated their potential as an activator of neurotrophic factor signalling via activation of the TrkB receptor, with emphasis on examining additional interactions with 7,8-DHF. The interactions of 7,8-DHF and several of its derivatives with the extracellular domain of TrkB receptor using a combination of molecular docking and dynamics tools were determined and presented here. Potential interactions of 7,8-DHF and its derivatives with VEGFR2 were also evaluated. Computational studies indicated 7,8-DHF to be an inhibitor of the VEGFR2. Effects of 7,8-DHF on photoreceptor cells in culture revealed that 7,8-DHF downregulated the VEGFR2 activity. Similar results were obtained in the *in vivo* study where 7,8-DHF administration led to a decrease in the activity of VEGFR2 in the retina. The combined *in silico*, cell culture and *in vivo* studies suggest emergence of 7,8-DHF as a dual action compound, which in addition to its known agonistic effects on TrkB receptor, can suppress the VEGFR2 activity.

## Supplementary Materials

Supplementary materials can be found at http://www.mdpi.com/1422-0067/16/09/21087/s1.
