# Supplementary material for: Exploring the Molecular Interactions of 7,8-Dihydroxyflavone and Its Derivatives with TrkB and VEGFR2 Proteins
Source: Int J Mol Sci. 2015 Sep 3;16(9):21087–108. doi: 10.3390/ijms160921087 (PMC4613243; doi:10.3390/ijms160921087)
Supplement: Supplementary file 1 [file ijms-16-21087-s001.pdf]

Supplementary Information

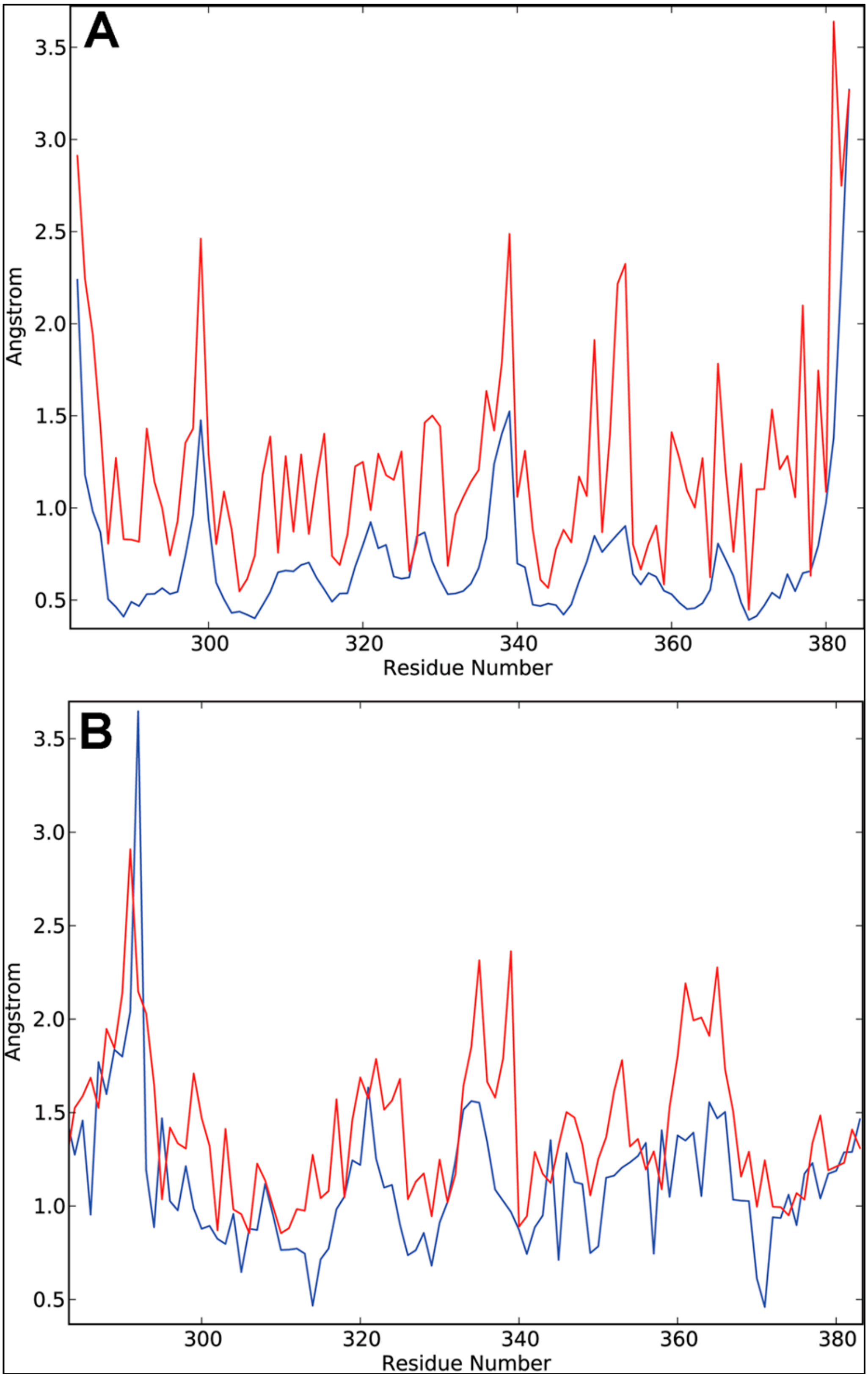

Figure S1. RMSF of backbone and side chain during 10 ns MD simulations. Blue color indicates backbone and red color indicates side chains. (**A**) 7,8-DHF-TrkB complex; and (**B**) 7,8-DHF-VEGFR2 complex.

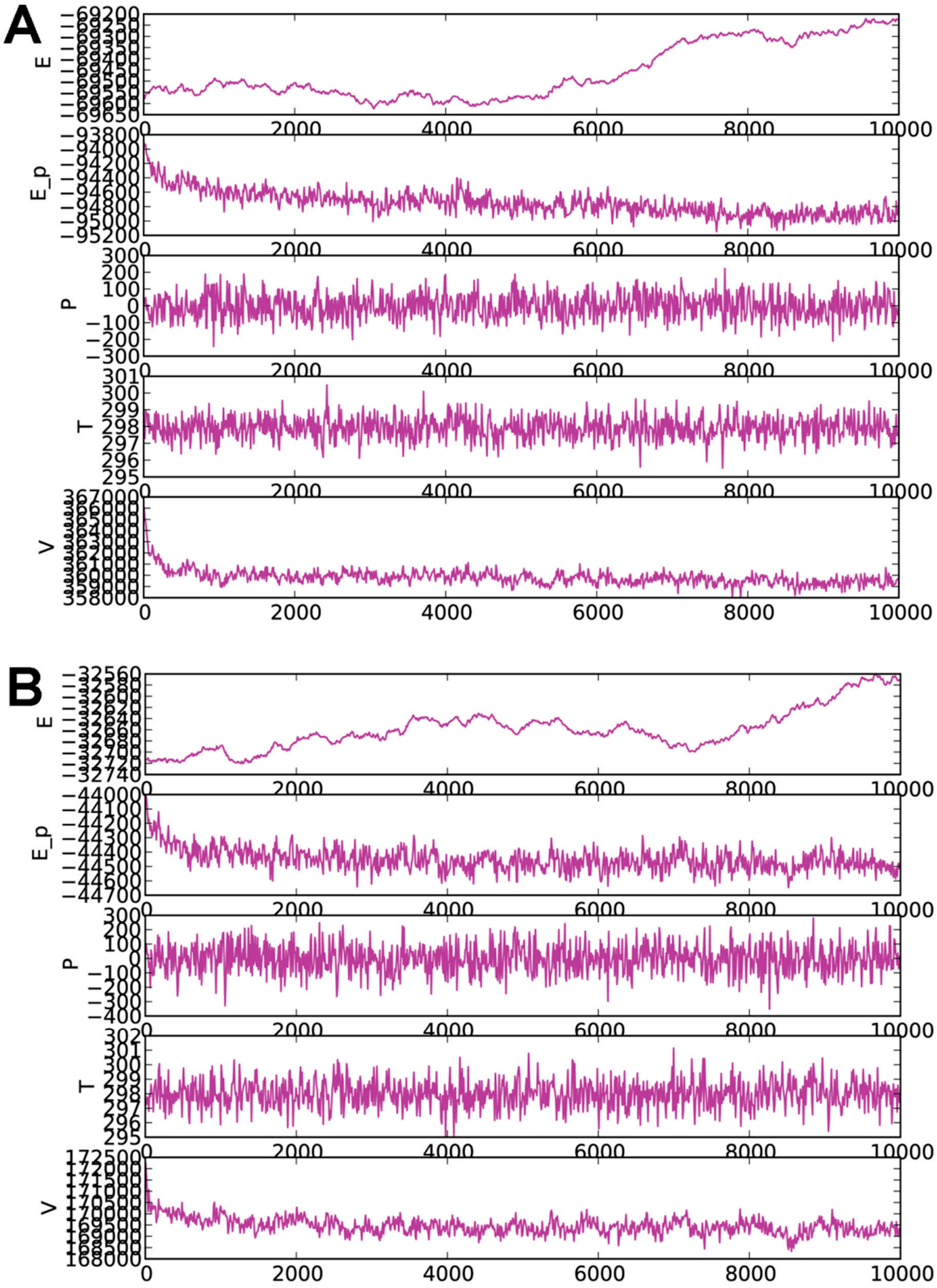

Figure S2. Energy peak during 10 ns MD simulation. (**A**) 7,8-DHF-TrkB complex; and (**B**) 7,8-DHF-VEGFR2 complex.

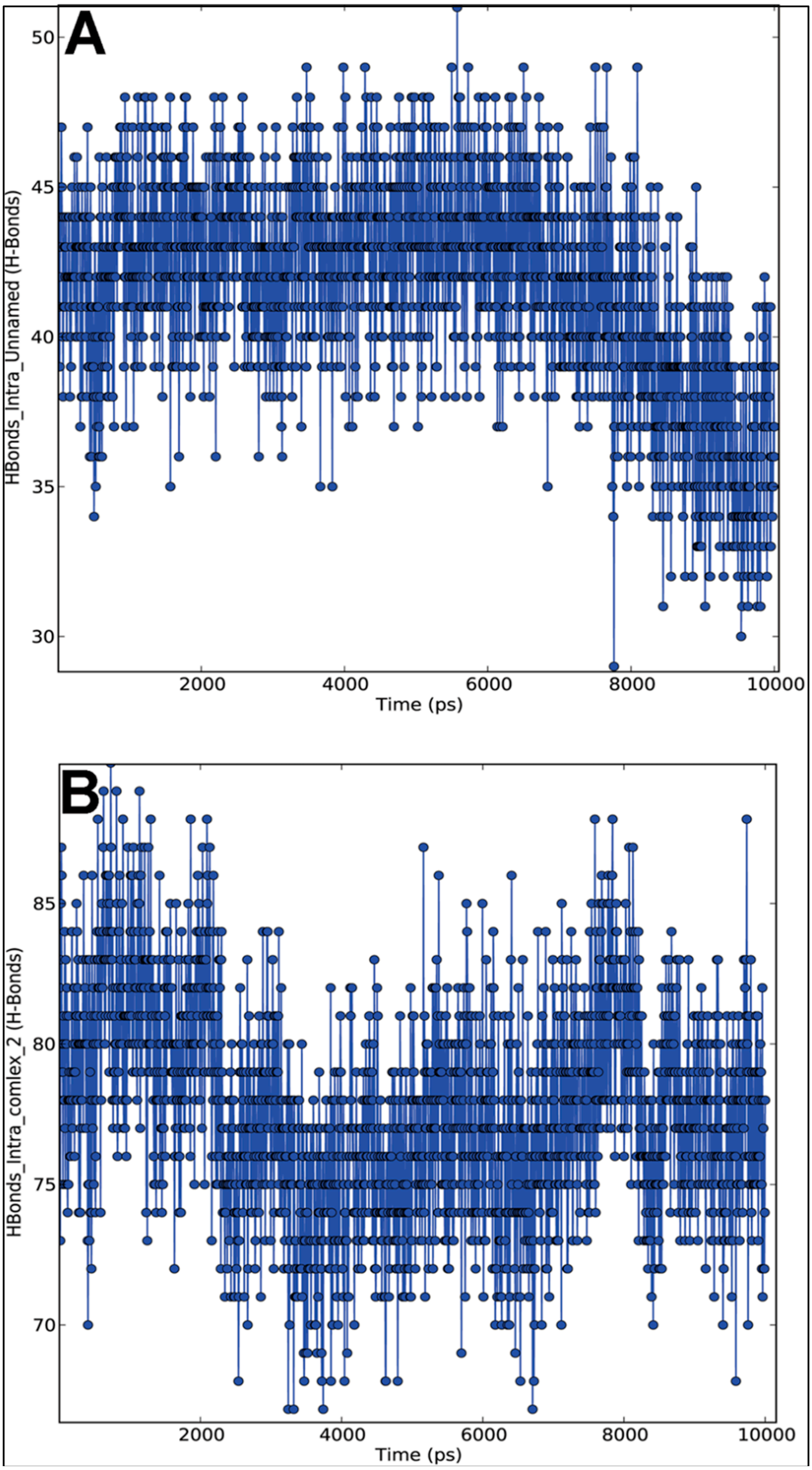

Figure S3. H-bonds formed during total course of 10,000 ps MD simulations. (**A**) 7,8-DHF-TrkB complex; and (**B**) 7,8-DHF-VEGFR2 docking complex.

A

```
sp|VEGFR2_HUMAN    MSELKILIHIGHHLNVVNLLGACTKPGGPLMVIVEFCKFGNLSTYLRSKR 932
sp|TrkB_HUMAN      QREAELLTMLQHQH-IVRFFGVCTE-GRPLLMVFEYMRHGDLNRFLRSHG 605
sp|IGF1R_HUMAN     LNEASVMKEFNCHH-VVRLLGVVSQ-GQPTLVIMELMTRGDLKSYLRSLR 1095
sp|STK4_HUMAN      MDSGTMVRAVGDEMGTVRVASTMTD-GANTMIEHDDTLPSQLGTMVINAE 373
sp|PK3CG_HUMAN     WLEFKCADPTALSNETIGIIFKHGDDLRQDMLILQILRIMESIWETESLD 861
sp|CDCP1_HUMAN     WNISVPRDQVACLTFFKERSGVVCQTGRAFMIIQEQRTRAEEIFSLDEDV 625
```

B **C**

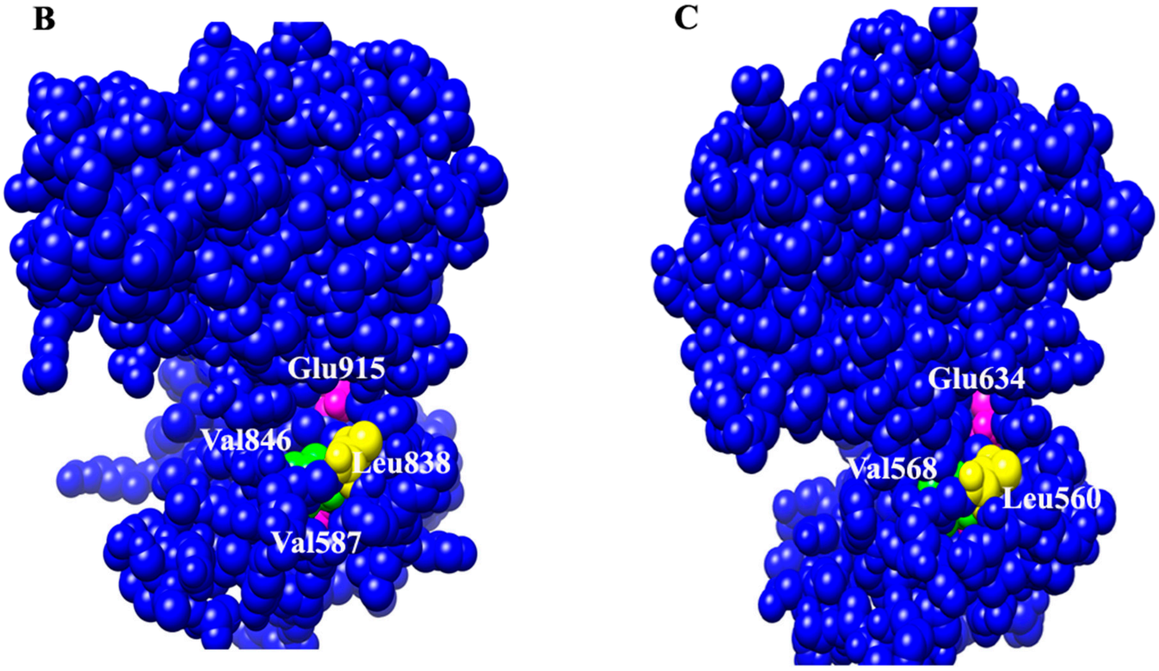

Figure S4. (**A**) Multiple sequence alignment of 7,8-DHF interacting residue at ATP binding site of VEGFR2 (red) with corresponding residues from various other kinases (blue and green); Space filled models (blue) highlighting the 7,8-DHF interacting residues of (**B**) VEGFR2 (**Left**) and its topological comparison with (**C**) TrkB (**Right**).

Table S1. Toxicity prediction of 37 di-hydroxy flavanoid derivatives including 7,8-DHF using ToxPredict program. S.No: Serial number; C.No.: Compound number; M_w: Molecular weight.

S.No	C.No	Pubchem CID	M_w	Carcinogenic Potency DBS Mouse	Carcinogenic Potency DBS MultiCellCall	Carcinogenic Potency DBS Mutagenicity	Carcinogenic Potency DBS Rat	Carcinogenic Potency DBS SingleCellCall	Eye Irritation
1	7,8-DHF	CID_44335549	256.25	non-carcinogen	non-carcinogen	non-mutagenic	non-carcinogen	non-carcinogen	No
2	C1	CID_17874507	256.25	non-carcinogen	non-carcinogen	non-mutagenic	non-carcinogen	non-carcinogen	No
3	C2	CID_676289	254.24	non-carcinogen	carcinogen	mutagenic	carcinogen	carcinogen	No
4	C3	CID_44257593	254.24	non-carcinogen	carcinogen	mutagenic	carcinogen	carcinogen	No
5	C4	CID_688660	254.24	non-carcinogen	carcinogen	mutagenic	non carcinogen	carcinogen	No
6	C5	CID_44257596	296.27	non-carcinogen	carcinogen	mutagenic	non carcinogen	carcinogen	No
7	C6	CID_165521	254.24	non-carcinogen	carcinogen	mutagenic	non carcinogen	carcinogen	No
8	C7	CID_455313	254.24	non-carcinogen	carcinogen	mutagenic	carcinogen	carcinogen	No
9	C8	CID_688715	254.24	non-carcinogen	carcinogen	mutagenic	carcinogen	carcinogen	No
10	C9	CID_145726	254.24	non-carcinogen	carcinogen	mutagenic	carcinogen	carcinogen	No
11	C10	CID_44257601	416.38	non-carcinogen	carcinogen	mutagenic	non-carcinogen	carcinogen	Yes
12	C11	CID_5315263	374.34	non-carcinogen	carcinogen	mutagenic	carcinogen	carcinogen	No
13	C12	CID_5393151	254.24	non-carcinogen	carcinogen	mutagenic	non-carcinogen	carcinogen	No
14	C13	CID_45933941	254.24	non-carcinogen	carcinogen	mutagenic	carcinogen	carcinogen	No
15	C14	CID_688659	254.24	non-carcinogen	carcinogen	mutagenic	non-carcinogen	carcinogen	No
16	C15	CID_5393152	254.24	non-carcinogen	carcinogen	mutagenic	non-carcinogen	carcinogen	No
17	C16	CID_182362	254.24	non-carcinogen	carcinogen	mutagenic	non-carcinogen	carcinogen	No
18	C17	CID_5282073	254.24	non-carcinogen	carcinogen	mutagenic	carcinogen	carcinogen	No
19	C18	CID_676030	254.24	non-carcinogen	carcinogen	mutagenic	non-carcinogen	carcinogen	No
20	C19	CID_5464381	314.29	non-carcinogen	carcinogen	mutagenic	non-carcinogen	carcinogen	No
21	C20	CID_14349487	254.24	non-carcinogen	carcinogen	mutagenic	non-carcinogen	carcinogen	No
22	C21	CID_5280442	284.26	non-carcinogen	carcinogen	mutagenic	non-carcinogen	carcinogen	No
23	C22	CID_5281607	254.24	non-carcinogen	carcinogen	mutagenic	non-carcinogen	carcinogen	No
24	C23	CID_5575368	358.34	non-carcinogen	non-carcinogen	non-mutagenic	non-carcinogen	non-carcinogen	No
25	C24	CID_5490092	416.38	non-carcinogen	carcinogen	mutagenic	carcinogen	carcinogen	Yes

Table S1. *Cont.*

S.No	C.No	Pubchem CID	M_w	Carcinogenic Potency DBS Mouse	Carcinogenic Potency DBS MultiCellCall	Carcinogenic Potency DBS Mutagenicity	Carcinogenic Potency DBS Rat	Carcinogenic Potency DBS SingleCellCall	Eye Irritation
26	C25	CID_11055	254.24	non-carcinogen	carcinogen	mutagenic	non-carcinogen	carcinogen	No
27	C26	CID_5353357	254.24	non-carcinogen	carcinogen	mutagenic	non-carcinogen	carcinogen	No
28	C27	CID_57368355	322.16	non-carcinogen	carcinogen	mutagenic	carcinogen	carcinogen	Yes
29	C28	CID_5318083	462.4	non-carcinogen	carcinogen	mutagenic	non-carcinogen	carcinogen	Yes
30	C29	CID_5391149	254.24	non-carcinogen	carcinogen	mutagenic	carcinogen	carcinogen	No
31	C30	CID_44257565	416.38	non-carcinogen	carcinogen	mutagenic	non-carcinogen	carcinogen	Yes
32	C31	CID_5391140	254.24	non-carcinogen	carcinogen	mutagenic	carcinogen	carcinogen	No
33	C32	CID_44257571	416.38	non-carcinogen	carcinogen	mutagenic	non-carcinogen	carcinogen	Yes
34	C33	CID_44257573	562.52	non-carcinogen	carcinogen	mutagenic	non-carcinogen	carcinogen	Yes
35	C34	CID_64982	446.36	non-carcinogen	non-carcinogen	mutagenic	non-carcinogen	carcinogen	Yes
36	C35	CID_14034267	254.24	non-carcinogen	carcinogen	mutagenic	non-carcinogen	carcinogen	No
37	C36	CID_44257569	416.38	non-carcinogen	non-carcinogen	non-mutagenic	non-carcinogen	non-carcinogen	Yes
